# SNP set analysis for detecting disease association using exon sequence data

**DOI:** 10.1186/1753-6561-5-S9-S91

**Published:** 2011-11-29

**Authors:** Ru Wang, Jie Peng, Pei Wang

**Affiliations:** 1 Department of Statistics, University of California, Davis, CA 95616, USA; 2 Division of Public Health Sciences, Fred Hutchinson Cancer Research Center, 1100 Fairview Avenue North, PO Box 19024, Seattle, WA 98109, USA

## Abstract

Rare variants are believed to play an important role in disease etiology. Recent advances in high-throughput sequencing technology enable investigators to systematically characterize the genetic effects of both common and rare variants. We introduce several approaches that simultaneously test the effects of common and rare variants within a single-nucleotide polymorphism (SNP) set based on logistic regression models and logistic kernel machine models. Gene-environment interactions and SNP-SNP interactions are also considered in some of these models. We illustrate the performance of these methods using the unrelated individuals data from Genetic Analysis Workshop 17. Three true disease genes (*FLT1*, *PIK3C3*, and *KDR*) were consistently selected using the proposed methods. In addition, compared to logistic regression models, the logistic kernel machine models were more powerful, presumably because they reduced the effective number of parameters through regularization. Our results also suggest that a screening step is effective in decreasing the number of false-positive findings, which is often a big concern for association studies.

## Background

High-throughput sequencing technologies have been evolving extraordinarily fast in the past few years. They have been recently applied to genome-wide association studies to study the effects of both common and rare variants. The different natures of these two types of variants call for distinct methods. For common variants, association tests based on individual SNPs are still widely used. However, such approaches suffer from multiple comparison problems and do not take into account possible interactions among variants. To overcome these limitations, analyses based on single-nucleotide polymorphism (SNP) sets have been developed to test the joint effect (either linear or nonlinear) of variants within a SNP set. For instance, Wu et al. [1] proposed a kernel-machine-based method for association studies; this approach is flexible for modeling various interactions and nonlinear effects. Mukhopadhyay et al. [2] derived similarity scores of genotypes between pairs of individuals using a kernel and then used these scores as the response variable in an analysis of variance (ANOVA) model to establish association between genotypes and phenotypes. Such methods tend to be more powerful and flexible than individual-SNP analysis.

Although many genome-wide association studies in the past focused on common variants, it is now widely believed that for complex diseases, rare variants are more likely to be functional than common variants [3]. Because rare variants usually have low marginal effects, multiple rare variants within a SNP set (e.g., a gene or a pathway) are often combined into a single variable to be used in tests for association. For example, Li and Leal [4] proposed a method for collapsing multiple rare variants into a single indicator that recorded whether or not the genome contained any rare variants for the SNP set under consideration; Madsen and Browning [5] proposed a weighted-sum score, where the weight for each variant indicator (0 for absent and 1 for present) was proportional to the inverse of its estimated standard deviation in the population. An overview of rare variant collapsing methods is provided by Dering et al. [6].

To effectively detect association signals, investigators might find it beneficial to jointly model the common and rare variants and to account for correlations among both variants. For this purpose, in this paper we introduce several methods to jointly model the common and rare variants within a SNP set. Note that throughout this paper SNPs with minor allele frequency (MAF) less than 1% are treated as rare variants and all other SNPs are treated as common variants.

We start with logistic regression models, including gene-environment interaction terms, and derive score statistics for testing the presence of any marginal or interaction effects. We then consider logistic kernel machine models, which can incorporate both interactions among SNPs and gene-environment interactions. This type of model is an extension of the method proposed by Wu et al. [1] and Liu et al. [7]. We also introduce a summary score for combining common variants based on the idea of principal fitted components [8], which is then used to reduce the dimensionality of the logistic regression model. We then use the 200 independently simulated data sets for unrelated individuals from Genetic Analysis Workshop 17 (GAW17) [9] to illustrate these methods, where a SNP set is defined as the observed SNPs (common and rare) within a gene. We also use a two-stage procedure, consisting of a screening stage and a testing stage, when analyzing the GAW17 data. The results suggest that the kernel machine methods enjoy better power than the score tests and that the screening stage helps to reduce the number of false-positive findings.

## Methods

### Logistic regression models and score tests

For the *i*th individual (*i* = 1, …, *n*), let response *y_i_* be 0 if the individual is unaffected and 1 if affected. Let *X_i_* be a *q* × 1 covariates vector (including an intercept term), *z_i_* be a *p* × 1 vector of SNP genotypes (or summary scores) for a given gene (SNP set) under testing, and *s_i_* be the environment covariate that is also included in *X_i_*. We consider the following logistic regression model with gene-environment interactions:(1)

where:(2)

The goal is to test the null hypothesis *H*_0_: *a* = *b* = 0, and we consider the corresponding score statistic. For a detailed derivation and expression of the score statistic, see Wang et al. [10].

### Logistic kernel machine models

Following Wu et al. [1] and Liu et al. [7], we now extend Eq. (1) to a semiparametric logistic regression model:(3)

where *h*(·) and *g*(·) belong to reproducing kernel Hilbert spaces *H_K_* and  generated by kernels *K*(·, ·) and  (·, ·), respectively. The penalized likelihoods *h*(·) and *g*(·) can be estimated by:(4)

Following Liu et al. [7], the solutions to Eq. (4) have the same form as the penalized quasi-likelihood estimators from the logistic mixed model:(5)

where ,  (i.i.d. stands for independent and identically distributed), , , and the *h_i_* and *g_i_* are independent. Denote *τ* = 1/*λ* and . Now, testing the null hypothesis of no genetic effects, *H*_0_: *h*(·) = *g*(·) = 0 in Eq. (3) can be reformulated as testing the absence of the variance components, *H*_0_: *τ* =  = 0 in model (5). As in Wu et al.’s [1] and Liu et al.’s [7] papers, we consider the (two-dimensional) test statistic:(6)

which is based on the score statistic of . The two components of Q* can be approximated by scaled chi-square distributions  and , respectively, through matching the means and variances [7].

Finally, we construct a combined test statistic:(7)

The corresponding *p*-value is then:(8)

where  is the cumulative distribution function of a chi-square distribution with *υ* degrees of freedom. For detailed derivations and expressions of Q*, , , , and , see Wang et al. [10]. Note that when both *K* and  are linear kernels, that is, , models (1) and (3) have the same form. However, they are treated differently, and consequently the corresponding test statistics are different.

### Summary score for common variants

For a gene with *p* common variants, we introduce the summary score:(9)

where *I_ik_* is the number of times the *k*th variant is observed in the *i*th individual,(10)(11)

and  and  are the number of times the *k*th variant is observed among affected and unaffected individuals, respectively, and *n^A^* and *n^U^* are the total numbers of affected and unaffected individuals, respectively. This summary score is derived based on the idea of principal fitted components for dimension reduction [8].

### Two-stage procedure

We propose a two-stage procedure to analyze the GAW17 data. In the screening stage, genes that do not show any statistical significance are filtered out. The main purpose of this stage is to achieve dimension reduction and at the same time to retain genes that are more likely to be associated with the disease. In the testing stage, we apply various methods to test the subset of genes that have passed the screening criteria.

In the screening stage, both genetic effects and gene-environment interaction effects are investigated, and common and rare variants are handled differently. Common variants are tested in the three subpopulations (Europeans, Asians, and Africans) separately, whereas rare variants are studied based on the whole population. For each gene, the genotypes of the common variants (coded 0, 1, or 2, denoting the number of minor alleles) are treated as a vector and the Hotelling *T*^2^ test is used to test whether there is a mean difference between the affected and unaffected individuals [4]. For rare variants, weighted-sum scores [5] are derived for the synonymous and nonsynonymous groups, denoted WS_syn_ and WS_nonsyn_, respectively. Then a two-dimensional Hotelling *T*^2^ test is performed based on WS_syn_ and WS_nonsyn_. To test gene-environment interactions, we consider the null hypothesis Corr(*G*, *E*|*Y* = 0) = Corr(*G*, *E*|*Y* = 1). We take the difference between Fisher’s *z* transformations of sample correlations for the affected and unaffected groups as the test statistic:(12)

Again, instead of testing each variant individually, we use combined scores for both common variants (Eq. (9)) and rare variants (the weighted-sum score) and test gene-environment interactions for each SNP set as a whole. In addition, for rare variants, we consider only the nonsynonymous variants.

In all the tests, the *p*-values are determined through permuting disease status (while keeping the total numbers of affected and unaffected individuals unchanged). Finally, genes are deemed to pass the screening and become candidates for the testing stage if they have (unadjusted) *p*-values smaller than a prespecified threshold (e.g., 0.1) for at least one of the tests.

In the testing stage, two kinds of models are considered: logistic regression models (Eq. (1)) and logistic kernel machine models (Eq. (5)). For all models, the covariates vector consists of Age, Sex, two principal component scores to account for population structure (see Results section for more details), and an environmental factor (Smoke status). For rare variants, we further introduce a combined weighted-sum score:

WS_combined_ = WS_syn_ + 2WS_nonsyn_, (13)

where nonsynonymous variants receive more weight.

For logistic regression models, we consider two different scenarios for the common variants, one using the original genotypes (referred to as logistic regression) and the other using the common score (Eq. (9)) with the weights calculated based on the corresponding screening data set (referred to as the logistic common score). In addition, WS_combined_ is used for both scenarios. Finally, score statistics are calculated and the *p*-values are determined using theoretical chi-square distributions.

For logistic kernel machine models (Eq. (5)), the original genotypes are used for common variants. We consider two different schemes for the kernels. One uses linear kernels for both *K* and , and the other uses a quadratic kernel for *K* that models interactions among variants and a linear kernel for . It is expected that the quadratic kernel will be more powerful if there are SNP-SNP interactions and that the linear kernel will be more powerful if such interactions are absent. For the quadratic kernel case, WS_combined_ is used and the method is referred to as the quadratic rare WS_combined_ method. For the linear kernel case, two scenarios are considered for combining rare variants, one using WS_combined_ (referred to as the linear rare WS_combined_ method) and the other using WS_nonsyn_ (referred to as the linear rare WS_nonsyn_ method). Moreover, for the kernel machine methods, the weighted-sum scores for rare variants and the genotypes of the common variants are both standardized (to have mean 0 and standard deviation 1) before model fitting.

In total, we consider five different methods in the testing stage, which are summarized in Table 1.

**Table 1:** Methods in the testing stage

Method	Model	Kernel	Common variants	Rare variants
Logistic regression	Logistic regression	NA	Genotypes	WS_combined_
Logistic common score	Logistic regression	NA	Common score	WS_combined_
Linear rare WS_combined_	Kernel machine	Linear	Genotypes	WS_combined_
Linear rare WS_nonsyn_	Kernel machine	Linear	Genotypes	WS_nonsyn_
Quadratic rare WS_combined_	Kernel machine	Quadratic	Genotypes	WS_combined_

## Results

### GAW17 data description

The GAW17 data we analyzed in this paper have 200 replicates, each consisting of data for 697 unrelated individuals. The genotypes, age, and sex of these individuals are from real studies and are kept fixed across the 200 replicates. One environmental risk factor (smoking status) and a binary disease status were simulated for each replicate [9]. Moreover, in all these replicates, the total numbers of affected and unaffected individuals are fixed to be 209 and 488, respectively, which reflects the population prevalence of this disease.

The 697 individuals were from seven different sources: Denver Chinese, Han Chinese, Japanese, Luhya, Yoruba, CEPH (European-descended residents of Utah), and Tuscan. Through principal components analysis on about 1,000 common variants (distance ≥ 50,000 bp) with MAF larger than 10%, the first two principal components clearly divide the sample into three distinct clusters, corresponding to Africans (Luhya and Yoruba), Asians (Chinese and Japanese), and Europeans (CEPH and Tuscan).

The genotype data consist of 24,487 SNPs from 3,205 genes on 22 autosomal chromosomes. The MAF for 74% of the SNPs is less than 1%. In our analysis, these are treated as rare variants, whereas all other SNPs are treated as common variants. Moreover, 2,208 genes contain at least one common variant, and the maximum number of common variants within a gene is 52. A total of 2,476 genes contain at least 1 rare variant and the maximum number is 179. One hundred sixty-two rare variants were removed from the analysis because they appeared in only one individual. Genes with a rare variant event occurring in less than 1% of individuals were removed, and 2,534 genes were left for subsequent analysis. Genotypes are coded as 0, 1, or 2, indicating the number of minor alleles at each locus.

### Findings

**Figure 1:**
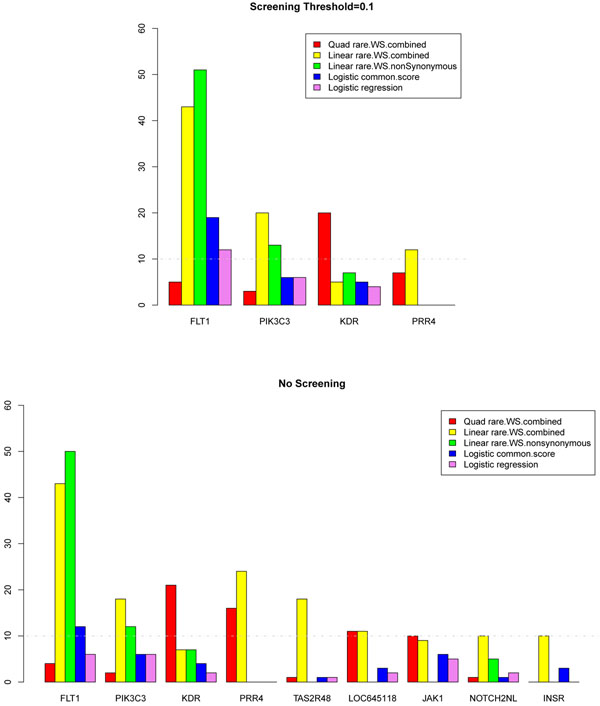
**Frequently selected genes and their selection frequencies**. For each gene, the height of the bar represents the number of times it has been selected across the 100 screening-testing pairs.

We randomly divided the 200 simulated replicates into 100 pairs. For each pair, one data set was used for screening and the other was used for testing. Across the 100 screening data sets, if a 0.1 threshold was used, the mean number of genes passing screening was 1,307 and 8 genes (*RUNX2*, *MUC3A*, *TMEM67*, *NIBP*, *AKAP2*, *GOLGA1*, *USP5*, and *FLT1*) were selected at least 95 times. If a 0.05 threshold was used, the mean number of genes passing screening was 824 and 1 gene (*FLT1*) was selected 95 times. For each pair of screening and testing data sets, genes that passed the screening step were tested using the five methods described in the Methods section. *P*-values were adjusted using the Holm procedure [11], which is an improvement of the Bonferroni procedure and controls the family-wise error rate. A gene was then said to be selected by a method if its corresponding adjusted *p*-value was less than 0.1. Throughout the 100 pairs of screening and testing data sets, if a threshold of 0.1 was used in the screening step, then four genes (*FLT1*, *PIK3C3*, *KDR*, and *PRR4*) were selected more than 10 times by at least one of the five testing methods. In contrast, if no screening was used (i.e., all 2,534 genes were passed to the testing stage), nine genes were selected more than 10 times by at least one of the five testing methods. The selection frequencies of these genes are illustrated in Figure 1.

As can be seen from Figure 1, *FLT1* was selected more than 40 times using the linear rare WS_combined_ method and more than 50 times using the linear rare WS_nonsyn_ method. Moreover, *PIK3C3* and *KDR* were selected about 20 times using the linear rare WS_combined_ method and the quadratic rare WS_combined_ method, respectively. Note that the quadratic kernel model is capable of capturing some of the SNP-SNP interaction effects, whereas the linear kernel model does not. Thus the fact that the quadratic kernel works better for *KDR* may imply that there are potential SNP-SNP interaction effects in this gene, which may result from the complicated disease model and/or correlation structure among the SNPs. Compared with the kernel machine methods, the two logistic regression methods gave less consistent results in terms of gene selection across the replicates. Furthermore, summarizing information of common variants by using the common score seemed to improve the power of the logistic regression model slightly.

Gene *FLT1* is on chromosome 13, and it contains 35 SNPs, of which 25 are rare variants. Applying the logistic regression model with gene-environment interaction (Eq. (1)) on the first replicate indicated that the (common) variant C13S523 was associated with disease status highly significantly (nominal *p* = 0.000817). This variant was nonsynonymous with a MAF of 6.7%. The weighted-sum score of the rare variants in *FLT1* also showed evidence of association (nominal *p* = 0.0033). Gene *KDR* is on chromosome 4 with 14 rare variants and 2 common variants. Gene *PIK3C3* has 7 variants (6 rare variants and 1 nonsynonymous common variant). It also seemed that this common variant was the reason that *PIK3C3* was picked by the linear rare WS_combined_ method about 20 times across the 100 replicates.

The results were obtained without knowledge of the underlying disease model. Afterward, we examined the GAW17 simulation model [9]. It turns out that, *FLT1*, *PIK3C3*, and *KDR* are true disease susceptible genes. However, other genes reported in Figure 1 were not directly related to disease status. By comparing the top and bottom panels in Figure 1, we see that the procedure with a screening step is effective in eliminating such genes. A closer look at the results reveals that these genes are mainly filtered by the screening step. For instance, *TAS2R48* was detected as a significant gene among 18 (out of 100) data pairs by the linear rare WS_combined_ method when no screening was applied. However, for 15 out of 18 pairs, *TAS2R48* would not pass the screening step if a 0.1 threshold was used.

## Conclusions

In this paper, we considered SNP set analysis for detecting disease-susceptible variants using exon sequence data. In large-scale association studies, there is often a need to combine information across variants to improve detection power. This is especially the case for rare variants. Here, we adopted the weighted-sum score of Madsen and Browning [5] to summarize information across rare variants within each SNP set. In addition, we proposed a summary score based on principal fitted components [8] to combine information across common variants. Moreover, the large number of variants poses challenges, such as multiple comparisons and modeling various interactions. To address this issue, we extended the logistic kernel machine methods used by Wu et al. [1] and Liu et al. [7] to include gene-environment interactions. Compared to logistic regression models, the logistic kernel machine models were more powerful, estimating the degrees of freedom in a data-adaptive way by accounting for correlations among the SNPs. Thus they reduced the effective number of parameters and consequently enjoyed improvements in power. Kernel machine models also had greater degrees of flexibility in modeling interactions and nonlinearity. We also applied a two-step procedure consisting of a screening stage and a testing stage to the GAW17 data. The results suggest that the screening stage is effective in decreasing the number of false-positive findings, which is often a big concern for association studies.

## Competing interests

The authors declare that there is/are no competing interests.

## Authors’ contributions

RW carried out data analysis and participated in the development of methods. JP and PW led the development of methods. All authors participated in drafting the manuscript. All authors read and approved the final manuscript.
